# Seroprevalence and biochemical parameters among patients with Chikungunya in Adamaoua region, Cameroon: A cross‐sectional study

**DOI:** 10.1002/puh2.157

**Published:** 2024-01-30

**Authors:** Lise Paule Djamko Toko, Borris Rosnay Galani Tietcheu, Ilyassa Dieudonné Tembar, Pascal Dieudonne Chuisseu Djamen, Nicolas Njintang Yanou

**Affiliations:** ^1^ Laboratory of Applied Biochemistry Department of Biological Sciences Faculty of Science The University of Ngaoundere Ngaoundere Adamaoua Cameroon; ^2^ Central Laboratory Ngaoundere Regional Hospital Ngaoundere Adamaoua Cameroon; ^3^ Higher Institute of Health Sciences Université des Montagnes Bangangté Cameroon

**Keywords:** Chikungunya virus, lipid profile, seroprevalence, transaminases

## Abstract

**Background:**

Chikungunya virus (CHIKV) infection is a vector‐borne febrile illness endemic to Africa. In Cameroon, few studies have documented human exposure to CHIKV, especially in the Adamaoua region. This study aimed to assess the CHIKV seroprevalence in Ngaoundere city and analyze likely association with social, clinical, and biochemical determinants.

**Methods:**

A cross‐sectional study was conducted at the Ngaoundere Regional Hospital from July to October 2022. The sociodemographic and clinical informations of participants were recorded using a standardized questionnaire. Then, blood tests were performed to determine the CHIKV status, the transaminase, and lipid profiles.

**Results:**

Of the 200 persons surveyed, 21.5% (43) were positive for anti‐CHIKV IgM antibodies. Age, sex, occupation, marital status, level of study, and place of residence were not significantly associated with CHIKV. However, fever (RR = 4.19; 42.86%; *p* = 0.0124), headaches (RR = 4.89; 50%; *p* = 0.0007), digestive disorders (RR = 6.52; 66.67%; *p* = 0.0001), and the presence of at least two clinical symptoms (RR = 2.55; 26.09%; *p* = 0.009) were significantly correlated with the presence of CHIKV compared with the absence of clinical symptoms (RR = 1, 10.23%). Similarly, cases of CHIKV were significantly more important in subjects with high aspartate aminotransferase (AST) than in those with normal AST (RR = 2.45; 37.50% vs. 15.28%; *p* = 0.0006). No significant association was found between alanine‐aminotransferase and lipid profile markers.

**Conclusion:**

Ngaoundere populations seem to be commonly infected with CHIKV, increasing the incidence of febrile symptoms and transaminase elevation. Clinical and metabolic monitoring is required.

## Graphical abstract

Chikungunya is an emerging arthrogenic arboviral disease in Cameroon, with limited reports of liver involvement. This study revealed a relatively high CHIKV seroprevalence (21.5%) among participants recruited in Ngaoundere (Cameroon). Symptoms, including fever, headaches, and digestive disorders, and high transaminase values, particularly AST levels, were significantly associated with CHIKV seropositivity.

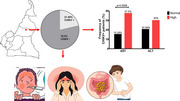

## INTRODUCTION

Arboviruses are viruses that survive in nature primarily through biological transmission between vertebrate hosts via hematophagous arthropods [1]. They are divided into four large families: Togaviridae, Flaviviridae, Bunyaviridae, and Reoviridae. The latter comes in several genera, all of which distributed across the globe, mainly in the tropics [2]. Arboviruses are spread by insect vectors, including mosquitoes, sandflies, and C*ulicinae* [3]. There are over 500 species of arboviruses, with approximately 100 of them being pathogenic to humans [4]. Dengue virus, yellow fever virus, *Zika virus*, *West Nile virus*, and Chikungunya virus (CHIKV) are the most common species worldwide [5].

CHIKV belongs to the Togaviridae family, genus *Alphavirus* [6]. It is enveloped with a single‐stranded, positive‐sense RNA genome. Its genome is 11.8 kb in size and has been evolutionarily classified into three genotypes: West African, East/Central/South African, African [7], and Asian [8]. CHIKV multiplies in the skin and spreads to the liver, muscles, joints, lymphoid tissues, and brain, most likely via the blood [9]. Symptoms associated with infection with this virus are joint pain before the fever, myalgia, headache, nausea, fatigue, and rash [10]. In symptomatic patients, CHIKV disease onset is typically 4–8 days (range 2–12 days) after the bite of an infected mosquito. It is characterized by an abrupt onset of fever, frequently accompanied by severe joint pain. During this phase, CHIKV can be detected through anti‐CHIKV IgM antibodies by rapid diagnostic or ELISA tests and by confirmation of the presence of viral RNA by RT‐PCR [11].

Chikungunya is an arboviral disease endemic to the African continent. First identified in Tanzania, it has spread to other countries around the world, including Kenya, India, and South East Asia [12]. In 2007, Europe was confronted with this disease, which was present in 20 of its countries [13]. In America, cases of CHIKV have been reported in approximately 1.1 million people, including death [14]. For decades, arbovirus diseases were thought to be minor causes of death and disability around the world. As a result, investing in arbovirus research has received low priority [15].

To date, very little information exists on the prevalence of CHIKV in Cameroon. Indeed, CHIKV was discovered during an outbreak of febrile syndrome in French military personnel in Douala and patients at a Yaoundé urban medical center in previous studies [16]. A previous study in Kumbo in the North‐West established a seroprevalence to anti‐CHIKV antibodies of 51.4% in the subjects surveyed and highlighted a predominance of larvae of *Aedes africanus* and *Culex* spp. in the sites sampled [17]. More recently, a molecular study detected this virus using a multiplex real‐time PCR in a Gabonese child seeking care in a health facility in Kyé‐ossi (South Cameroon) [18]. Other studies have even discovered a new clade known as the Central African clade, which has essential mutations on the E1 and E2 glycoproteins that confer infectious potential [7]. In addition, some studies have reported a prevalence of 4.5% and 12.6% in Yaoundé and Dizangué, respectively [19].

Furthermore, previous research in the city of Ngaoundere (Adamaoua, Cameroon) has revealed the presence of vector insects from the Culicinae subfamily at 90.11%, particularly in the locality of Dang [20]. This suggests that populations of this locality live in a risky environment for CHIKV transmission. Despite the abundance of these vectors and the growing prevalence of CHIKV in Cameroon, cases of CHIKV have not yet been reported in Ngaoundere, to the best of our knowledge. Furthermore, clinical investigations showing the presence of liver disorders in chikungunya patients are still lacking. This study aimed to determine CHIKV seroprevalence in Ngaoundere, examine the association between seropositivity and clinical and sociodemographic factors, and assess the effect on biochemical parameters of liver function.

## METHODS

### Study design, setting, and population

This cross‐sectional and descriptive study was carried out from July 15 to October 15, 2022, at the Ngaoundere Regional Hospital, which is a tertiary care hospital located in the Ngaoundere 1 district of the Adamaoua region. Ngaoundere is the capital of both the Adamaoua region and the Vina division. It is also a crossroad city between southern and northern Cameroon, having a population estimated at 300,000 inhabitants [21]. Its climate includes a hot season from December to March and a wet season from April to November. The yearly rainfall is 2248 mm. According to current data, temperatures vary from 17 to 34°C, whereas relative humidity ranges from 22% to 89% [22].

Cameroonian subjects of both sexes, aged at least 18, with febrile or non‐febrile symptoms, no history of hepatitis, and no signs of dyslipidemia, and referred to the Central Laboratory of the Ngaoundere Regional Hospital for a biological examination, were included. Subjects with a history of liver disease, alcoholics, and diabetics were not admitted in this study. Moreover, children, individuals who were unable to offer permission, and pregnant women were excluded.

The minimum sample size calculated was 169 using the CHIKV seroprevalence in the city of Dizangue (12.6%) [19] as a reference prevalence. However, to ensure a representative sample, 200 participants were recruited.

### Study variables

We started from the hypothesis that the presence of insect vectors like Culicinae in Ngaoundere, as shown by earlier reports [20], increases the risk for CHIKV transmission in humans. Therefore, our primary outcome measure was to highlight the exposure of participants to CHIKV. We did it via serological detection of anti‐CHIKV antibodies by immunochromatography using rapid diagnostic tests.

Considering that the presence of the CHIKV virus may be followed by liver abnormalities, we hypothesized that liver‐related biochemical parameters might be significantly altered in CHIKV+‐positive patients in comparison with the non‐positive group. As a result, transaminases and lipid parameters were quantified as secondary outcome measures in both groups using colorimetric methods.

### Sample collection and preparation

Participants were welcomed and informed about the study's goals, analyses to be performed, risks, and benefits upon arrival at the Ngaoundere Regional Hospital's central laboratory. An informed consent form was obtained and signed. A paper‐based, structured questionnaire was administered to gather information on sociodemographic, and clinical parameters. A sterile syringe was then used to collect 4 mL of blood from the elbow crease. The blood was transferred to a dry tube and centrifuged at 3000 rpm for 5 min in a tabletop centrifuge. The serum obtained was either used immediately for CHIKV diagnosis or stored for 1 week at 2–8°C for biochemical assays. All biochemical data were recorded on the survey form.

### Serological diagnosis with Chikungunya virus and biochemical assays

Serum samples were screened for the presence of anti‐CHIKV IgM antibodies using the rapid diagnostic kits provided by Encode Medical (HK) Limited and following the manufacturer's instructions. This antibody test is a lateral flow immunochromatographic test based on the principle of IgM capture. It involves a burgundy‐colored conjugate pad containing colloidal gold‐conjugated CHIKV antigens and rabbit IgG‐gold conjugates, and a nitrocellulose membrane strip with a side band (T band) and a control band (C band). When the specimen is dispensed, anti‐CHIKV antibodies bind to CHIKV conjugates, forming an immune complex that is captured by the pre‐coated anti‐human IgG antibody, indicating a positive result. The test has a relative sensitivity of 90.3% and a specificity of 100%.

Total cholesterol (TC), high‐density lipoprotein cholesterol (HDL‐C), low‐density lipoprotein cholesterol (LDL‐C), and triglycerides (TG) levels were measured using a lipid analyzer (LipiDiag) linked to a blood lipid test strip (MSL‐301) provided by Medsinglong Global Group Co., Ltd. and under the manufacturer's instructions, as previously described [23]. Alanine‐aminotransferase (ALT) and aspartate aminotransferase (AST) were assayed using commercial kits from Biolabo, following the manufacturer's instructions.

### Statistical analysis

The data collected were coded and inserted into Sphinx software to create a database. These data were then analyzed with GraphPad Prism 8.0.1 software. The Chi‐square test examined the association between the disease and sociodemographic, clinical, and biochemical factors. Relative risks (RR) and the 95% confidence intervals were determined. The Mann–Whitney test was used to compare means of biochemical parameters between CHIKV seropositive and seronegative patients. The significance level was set at *p* < 0.05.

## RESULTS

Of the 200 participants surveyed, 21.5% (43) were tested positive for CHIKV antibodies (Table 1).

**TABLE 1:** Statistical association between sociodemographic factors and chikungunya seropositivity in the surveyed participants at the Ngaoundere Regional Hospital, Adamaoua, Cameroon from July to October 2022.

		*Total*	*CHIKV+*	*F(%)*	*Risk ratio*	*Confidence interval*	*p‐Value*
***Gender***	*Male*	*68*	*15*	*22.06*	*1.04*	*0.59–1.77*	*0.89*
	*Female*	*132*	*28*	*21.21*	*1.00*		
***Age (years)***	*18–30*	*80*	*17*	*21.25*	*1.56*	*0.56–4.77*	*0.42*
	*30–40*	*39*	*11*	*28.21*	*2.07*	*0.72–6.48*	*0.19*
	*40–50*	*35*	*7*	*20.00*	*1.47*	*0.46–4.8*	*0.53*
	*50–60*	*22*	*3*	*13.64*	*1.00*		
	*60–70*	*19*	*4*	*21.05*	*1.54*	*0.43–5.59*	*0.52*
	*70–80*	*5*	*1*	*20.00*	*1.47*	*0.22–7.69*	*0.71*
***Study level***	*None*	*16*	*5*	*31.25*	*1.65*	*0.67–3.55*	*0.27*
	*Primary*	*34*	*8*	*23.53*	*1.24*	*0.57–2.54*	*0.58*
	*Secondary*	*71*	*15*	*21.13*	*1.11*	*0.59–2.09*	*0.74*
	*University*	*79*	*15*	*18.99*	*1.00*		
***Profession***	*Informal*	*95*	*21*	*22.11*	*1.22*	*0.41–4.48*	*0.76*
	*Formal*	*56*	*12*	*21.43*	*1.18*	*0.38–4.48*	*0.8*
	*Students*	*38*	*8*	*21.05*	*1.16*	*0.34–4.55*	*0.83*
	*Retired*	*11*	*2*	*18.18*	*1.00*		
***Matrimonial status***	*Single*	*72*	*13*	*18.06*	*1.00*		
	*Married*	*128*	*30*	*23.44*	*1.30*	*0.74–2.33*	*0.37*
***Place of residence***	*Ngaoundere 1*	*106*	*29*	*27.36*	*3.56*	*0.78–20.30*	*0.12*
	*Ngaoundere 2*	*81*	*13*	*16.05*	*2.09*	*0.43–12.23*	*0.43*
	*Ngaoundere 3*	*13*	*1*	*7.69*	*1.00*		

Abbreviation: CHIKV, Chikungunya virus.

As shown by Table 1, the population is made up of more women than men, with a sex ratio F/M of 1.94. Young people are the most represented with an average age of 37.89 years. The majority of respondents are between the ages of 18 and 29, followed by those between the ages of 30 and 39. Students account for 39.59% of the sample, with pupils accounting for 36.04%. The informal sector has the highest number of participants, followed by the formal sector. The majority of those polled are married, with singles coming in second. People who have been divorced or widowed, on the other hand, are not present.

In terms of clinical history, only 0.5% of the sample was HIV‐positive, and 16.5% reported being on medication before the study (Table 2). Clinical symptoms were discovered in most of the participants. Those with at least two clinical symptoms were the most represented. Muscle pain was the most common clinical sign among subjects with only one clinical symptom, followed by headaches.

**TABLE 2:** Statistical association between clinical antecedents and chikungunya seropositivity in the surveyed participants at the Ngaoundere Regional Hospital, Adamaoua, Cameroon, from July to October 2022.

		*Total*	*CHIKV+*	*F (%)*	*Risk ratio*	*Confidence interval*	*p‐Value*
***HIV serology***	*Positive*	*1*	*0*	*0*	*1*		
	*Negative*	*199*	*43*	*21.61*	*∞*	*0.25–∞*	*0.59*
***Types of symptoms***	*Fever*	*7*	*3*	*42.86*	*4.19*	*1.34–10.34*	***0.0124*** ^*^
	*Headaches*	*10*	*5*	*50*	*4.89*	*1.91–10.88*	***0.0007*** ^***^
	*Muscle pain*	*20*	*4*	*20*	*1.96*	*0.67–5.22*	*0.22*
	*Digestive disorders*	*6*	*4*	*66.67*	*6.52*	*2.47–13.82*	***0.0001*** ^***^
	*≥2 symptoms*	*69*	*18*	*26.09*	*2.55*	*1.24–5.25*	***0.009*** ^***^
	*None*	*88*	*9*	*10.23*	*1*		
***Past medication***	*Yes*	*33*	*8*	*24.24*	*1.16*	*0.57–2.14*	*0.67*
	*No*	*167*	*35*	*20.96*	*1*		

Abbreviation: CHIKV, Chikungunya virus.

* *p* < 0.05.

*** *p* < 0.001 using the Chi‐square test.

### Predictors of the CHIKV seropositivity

Table 1 shows the statistical association between different sociodemographic parameters and CHIKV seropositivity in the surveyed group. None of the sociodemographic parameters studied are significantly associated with the presence of CHIKV in the subjects surveyed. However, subjects aged 30–40 seem more exposed than those aged 50–60, even if *p* > 0.05. Similarly, subjects coming from Ngaoundere I and Ngaoundere II seem to be more at risk for CHIKV than those from Ngaoundere III although this risk is not significant.

As far as clinical profile is concerned, HIV serology and drug intake were not significantly correlated with the presence of CHIKV, unlike the type of clinical symptoms (Table 2). Indeed, fever, headaches, digestive disorders, and the presence of at least two clinical symptoms were significantly associated with the presence of the virus than the absence of clinical symptoms.

Regarding the biochemical parameters, Table 3 shows the average values of transaminases and lipid markers obtained in the sample. Participants have overall normal biochemical values. However, the most dispersed were the ALT values followed by AST and TG, whereas the least dispersed were the TC values. Considering the dispersion of these data, we also wanted to probe the profile of biochemical abnormalities within the studied group (Table 4). It emerges that 6.5% of subjects have total hypocholesterolemia, 5.5% LDL hypercholesterolemia, 0.5% HDL hypocholesterolemia, and 27.5% high cardiovascular risk. Regarding transaminases, 25% of subjects have abnormally high AST values against 10% for ALT. Then, the association of these parameters with CHIKV was evaluated, as shown in Table 5. No statistical link was found between CHIKV seropositivity and ALT values. However, AST was significantly correlated with the presence of CHIKV with much higher cases of CHIKV seropositivity in those with high AST than in those with normal AST. Regarding lipid profile parameters, no significant association was noted with HDL‐C, LDL‐C, TC, and TC/HDL‐C. The comparative analysis of serum concentrations of different biochemical markers between CHIKV+ and CHIKV− patients presented in Table 6 also shows significantly higher AST mean levels in seropositive than seronegative patients.

**TABLE 3:** Mean values of some biochemical parameters within the surveyed participants at the Ngaoundere Regional Hospital, Adamaoua, Cameroon, from July to October 2022.

		*Means ± SEM*	*Reference values*	*Range (min/max)*		*Coefficient of variation (%)*
***Lipid markers***	*TC (mg/dL)*	*141.9 ± 2.68*	*100–400*	*70*	*279*	*26.75*
	*HDL‐C (mg/dL)*	*36.24 ± 0.93*	*15–100*	*10*	*82*	*36.49*
	*LDL‐C (mg/dL)*	*80.96 ± 2.30*	*<135*	*7.52*	*206*	*40.2*
	*TG (mg/dL)*	*106.9 ± 4.43*	*45–500*	*44*	*490*	*58.63*
	*TC/HDL*	*4.39 ± 0.14*	*[0–5[*	*1.77*	*12.64*	*45.36*
***Transaminases***	*AST (IU/L)*	*26.55 ± 1.48*	*H ≤ 41; F ≤ 31*	*3.56*	*233*	*78.97*
	*ALT (IU/L)*	*18.46 ± 1.24*	*H ≤ 38; F ≤ 31*	*3*	*194*	*95.35*
	*AST/ALT*	*1.43*				

Abbreviations: ALT, alanine‐aminotransferase; AST, aspartate aminotransferase; HDL‐C, high‐density lipoprotein cholesterol; LDL‐C, low‐density lipoprotein cholesterol; TC, total cholesterol; TG, triglycerides.

**TABLE 4:** Frequencies of biochemical abnormalities within the surveyed participants at the Ngaoundere Regional Hospital, Adamaoua, Cameroon, from July to October 2022.

		*Hypo*		*Normal*		*Hyper*	
		*N*	*%*	*N*	*%*	*N*	*%*
***Lipid markers***	*TC (mg/dL)*	*13*	***6.5***	*187*	*93.5*	*0*	*0*
	*HDL‐C (mg/dL)*	***1***	***0.5***	*199*	*99.5*	*0*	*0*
	*LDL‐C (mg/dL)*	*0*	*0*	*189*	*94.5*	***11***	***5.5***
	*TG (mg/dL)*	*0*	*0*	*200*	*100*	*0*	*0*
	*TC/HDL*	*0*	*0*	*145*	*72.5*	***55***	***27.5***
***Transaminases***	*AST (IU/L)*	*0*	*0*	*150*	*75*	***50***	***25***
	*ALT (IU/L)*	*0*	*0*	*180*	*90*	***20***	***10***

Abbreviations: ALT, alanine‐aminotransferase; AST, aspartate aminotransferase; HDL‐C, high‐density lipoprotein cholesterol; LDL‐C, low‐density lipoprotein cholesterol; TC, total cholesterol; TG, triglycerides.

**TABLE 5:** Statistical association between biochemical profile and chikungunya seropositivity in the surveyed participants at the Ngaoundere Regional Hospital, Adamaoua, Cameroon, from July to October 2022.

			*Total*	*CHIKV+*	*F (%)*	*RR*	*Confidence interval*	*p‐*Value
***Transaminases***	*ALT*	*Normal*	*180*	*37*	*20.56*	*1.00*		
		*High*	*20*	*6*	*30.00*	*1.45*	*0.67–2.75*	*0.32*
	*AST*	*Normal*	*144*	*22*	*15.28*	*1.00*		
	*High*	*56*	*21*	*37.50*	*2.45*	*1.46–4.05*	***0.0006*** ^***^
***Lipid profile***	*TC*	*Normal*	*186*	*40*	*21.51*	*1.00*	*0.42–2.92*	*0.99*
		*Low*	*14*	*3*	*21.43*	*1.00*		
	*HDL‐C*	*Normal*	*100*	*19*	*19.00*	*1.00*		
		*High*	*100*	*24*	*24.00*	*1.26*	*0.74–2.15*	*0.38*
	*LDL‐C*	*Normal*	*8*	*3*	*37.50*	*1.80*	*0.63–3.64*	*0.26*
		*High*	*192*	*40*	*20.83*	*1.00*		
	*TG*	*Normal*	*169*	*33*	*19.53*	*1.00*		
		*High*	*31*	*10*	*32.26*	*1.65*	*0.88–2.85*	*0.11*
	*TC/HDL‐C*	*Normal*	*107*	*19*	*17.76*	*1.00*		
		*High*	*93*	*24*	*25.81*	*1.45*	*0.85–2.47*	*0.16*

Abbreviations: ALT, alanine‐aminotransferase; AST, aspartate aminotransferase; CHIKV, Chikungunya virus; HDL‐C, high‐density lipoprotein cholesterol; LDL‐C, low‐density lipoprotein cholesterol; RR, relative risks; TC, total cholesterol; TG, triglycerides.

*** *p* < 0.001 using the Chi‐square test.

**TABLE 6:** Comparison of serum concentrations of different biochemical markers between Chikungunya virus (CHIKV)‐positive and CHIKV‐negative patients sampled at the Ngaoundere Regional Hospital, Adamaoua, Cameroon, from July to October 2022.

*Parameters*	*CHIKV+*	*CHIKV−*	*p‐Value*
*ALT (IU/L)*	*20.73 ± 2.73*	*17.83 ± 1.39*	*0.36*
*AST (IU/L)*	*31.05 ± 2.66*	*25.32 ± 1.73*	0.012*
*AST/ALT*	*2.37 ± 0.35*	*1.94 ± 0.11*	*0.7*
*TC (mg/dL)*	*139.56 ± 5.51*	*142.58 ± 3.07*	*0.81*
*HDL (mg/dL)*	*33.18 ± 1.77*	*37.08 ± 1.01*	*0.11*
*LDL (mg/dL)*	*80.08 ± 4.83*	*81.2 ± 2.62*	*0.91*
*TG (mg/dL)*	*118.06 ± 9.20*	*103.88 ± 5.04*	*0.075*
*TC/HDL*	*4.68 ± 0.32*	*4.3 ± 0.15*	*0.19*

Abbreviations: ALT, alanine‐aminotransferase; AST, aspartate aminotransferase; CHIKV, Chikungunya virus; HDL, high‐density lipoprotein; LDL, low‐density lipoprotein; TC, total cholesterol; TG, triglycerides.

*p < 0.05 using the Mann–Whitney test.

## DISCUSSION

The prevalence of anti‐CHIKV IgM antibodies discovered through serological testing is 21.5%. IgM was tested because it gives information on a recent infection. To the best of our knowledge, this is a novel finding that suggests Ngaoundere populations are highly susceptible to CHIKV. This also confirms active arbovirus transmission in this area. This observation is backed by a recent Cameroonian investigation that found mosquitos of the genus *Aedes* in the city of Ngaoundere [20]. Furthermore, the seroprevalence is higher than that observed in Yaoundé (4.5%) and Dizangué (12.6%) [19]. The difference could lie in the study period. Indeed, our study was conducted primarily during the rainy season, whereas the other authors’ study was conducted throughout the year, including during the dry season, which is known for having a low prevalence of mosquitoes of the genus *Aedes*, thus diluting the risks of transmission and the overall prevalence. Some authors showed a higher density of *Aedes* mosquitoes in Yaoundé during the rainy season (4706) than during the dry season (1626) [24]. Furthermore, the geographical location of Ngaoundere and the study period, which corresponds to school holidays, raise the risk of introducing viraemic patients due to the high mobility of inhabitants [4]. Our results, however, are lower than those of a Pakistani study (70%) [25]. The difference could be due to the larger sample size in the other studies, but it could also be due to the different specificity of the RDT kit used to detect IgM or the use of more sensitive detection methods, such as PCR.

Regarding sociodemographic parameters, no significant association was observed between gender, age, level of education, occupation, marital status, place of residence, and CHIKV seropositivity. However, people in the age group of 30–40 years had a higher RR than those in 50–60 years. This corroborates a Mexican study that reported a mean age of 37.2 and 39.2 years in viremic and post‐viremic patients with CHIKV [26]. The most affected age groups here are 31–40 and 41–50 years. This also agrees with a Pakistani study that highlighted an average age of 31.8 years among CHIKV‐positive subjects in Pakistan [25]. Therefore, the lack of herd immunity may play a role in their vulnerability. Contrary to our results, some studies, particularly in French Polynesia, suggest that women are more exposed to CHIKV infection than men [27]. Even if the reasons for this exposure are still poorly understood, some authors indicate that estradiol, produced by women, would increase their tendency to produce antibodies more than men [28]. Furthermore, other sources reveal that estrogen receptor modulators have the same protective effect against alphaviruses by acting as replication inhibitors [29].

Regarding clinical history, symptoms, such as fever, headache, and digestive disorders, were significantly associated with the presence of CHIKV compared with the absence of clinical symptoms. This finding indicates that CHIKV infection is primarily symptomatic in our study and thus active. This result is similar to previous reports that indicated that in a rural community in Brazil, 54.2% of HIV‐positive CHIKV patients had a fever and joint pain for more than 2 years and the infection was symptomatic in 40.7% [30]. It is also consistent with previous research from India [31] and Nigeria [32], which showed that serological detection of the virus is associated with the occurrence of a variety of clinical symptoms such as fever and joint pain. Similarly, another study in Mexico discovered that fever (100%), headache (82.3%), digestive disorders (46.8%), and polyarthritis (100%) were symptoms associated with the presence of CHIKV in surveyed patients [33]. However, as patients presenting with isolated symptoms (fever, headache, and digestive disorders) are very few, less than 10 per group, a possible risk of overestimating the correlation with positive RDT should not be ruled out.

Among transaminases, AST specifically was significantly associated with the presence of CHIKV, whereas ALT's RR was increased but not significantly. A retrospective study in Thailand demonstrated that the presence of CHIKV infections in adult patients caused a mild‐to‐moderate increase in ALT [34]. Indeed, although ALT averages between CHIKV+ and CHIKV− patients are comparable in our study, AST means are significantly higher in CHIKV+ patients than in CHIKV− patients (*p* < 0.05). This finding, along with previous reports, suggests that although the presence of the virus increases the risk of liver damage, injury to other target organs such as the heart and muscles is greater [33]. CHIKV has a strong proclivity to infect tissues, such as the spleen, bones, heart, lungs, and skin [35], leading to organ dysfunction and blood AST elevations. In some studies, the high AST levels were positively correlated with high prevalences of arthralgia in children [11]. This finding is consistent with those of a previous study that recently reported significant liver abnormalities in subjects with arthritic chikungunya compared to those without [36]. These abnormalities were manifested by a significant increase in AST, ALT, the AST/ALT ratio, and bilirubin mean concentrations. This result could also be explained by taking paracetamol, ibuprofen, or diclofenac to relieve CHIKV symptoms because these nonsteroid anti‐inflammatory drugs can cause biochemical changes such as increased transaminases [37]. It would have been critical to diagnose the markers of arthritis in this work to determine whether or not the CHIKV+ patients are arthritic. Similarly, no statistically significant link was noted between CHIKV seropositivity and lipid profile abnormalities. This is consistent with previous investigations, which discovered that CHIKV patients had normal triglyceride and cholesterol levels [27, 38].

## CONCLUSION

This study reports a CHIKV seroprevalence of 21.5% in Ngaoundere populations (Adamaoua, Cameroon), hence demonstrating an active transmission of this virus by insect vectors in this locality. Additionally, this finding might point out the existence of a possible source of infection with the potential to spread to nearby regions. The majority of seropositive subjects experienced fever symptoms and abnormally high AST levels, suggesting that chikungunya might affect the liver, joints, and other vital organs in these populations. Chikungunya clinical surveillance is required in the Adamaoua region to forecast outbreaks and related consequences.

## AUTHOR CONTRIBUTIONS

*Conceptualization; data curation; methodology; formal analysis; supervision; writing—writing—review and editing*: Borris Rosnay Galani Tietcheu. *Investigation, methodology; formal analysis; writing—original draft*: Lise Paule Djamko Toko. *Methodology; formal analysis; writing—review*: Ilyassa Dieudonné Tembar, Pascal Dieudonne Chuisseu Djamen, and Nicolas Njintang Yanou.

## CONFLICT OF INTEREST STATEMENT

The authors have no conflicts of interest to report concerning this paper.

## ETHICS STATEMENT

A research permit (N°608/AR/RA/DSP/BFP/NGE dated July 18, 2022) was granted by Adamaoua's Regional Delegation for Public Health and the Ethics Committee of the Ngaoundere Regional Hospital. Before taking part in the study, all participants were given a thorough explanation of the objectives, benefits, and risks in French and/or Fulfulde languages. All participants read and signed a written informed consent form. The procedures were carried out in line with the standards of the committee in charge of human experimentation (institutional or regional) as well as the World Medical Association's Helsinki Declaration (1964, most recently amended in 2008).

## Data Availability

The data that support the findings of this study are available from the corresponding author upon reasonable request.
